# Recent advances of kiwifruit genome and genetic transformation

**DOI:** 10.1186/s43897-024-00096-1

**Published:** 2024-05-10

**Authors:** Yingzhen Wang, Yongsheng Liu

**Affiliations:** School of Horticulture, Anhui Agricultural University, Hefei, 230036 P. R. China

Native to China, kiwifruit (*Actinidia ssp.*) is one of the most successfully domesticated fruit tree species in the 20th century. This genus encompasses 54 species (75 taxonomic units), wherein *A. chinensis*, *A. deliciosa*, *A. arguta* and *A. eriantha* are currently cultivated. Thanks to the rapid development of sequencing/gene-editing technologies and molecular biology, intensive investigations on kiwifruit genomics and functional genomics have been carried out. The draft genome of the *A. chinensis* cv. ‘Hongyang’ was initially released in 2013 (Huang et al. 2013) and the genome quality of this species has been updated by several investigations (Pilkington et al. 2018; Wu et al. 2019; Han et al. 2023; Yue et al. 2023). Subsequently, a number of *Actinidia* genomes including *A. eriantha* (Tang et al. 2019; Yao et al. 2022; Liao et al. 2023; Wang et al. 2023a), A. *chinensis* var. *deliciosa* (Liu et al. 2023a; Xia et al. 2023), A. *hemsleyana* (Yu et al. 2023), A. *arguta* (Akagi et al. 2023), A. *polygama* (Akagi et al. 2023), A. *rufa* (Akagi et al. 2023) and *A.* *latifolia* (Han et al. 2023) were successively deciphered. Meanwhile, Yue et al. (2020) established the kiwifruit genome database (http://kiwifruitgenome.org/), integrating various omics data including genomics and transcriptomics, which provides a comprehensive platform for kiwifruit research community. Based on these high-quality assembled genomes, the regulatory mechanisms underlying various horticulturally important traits, such as kiwifruit sex determination (Akagi et al. 2018, 2019, 2023; Yue et al. 2024), flowering time (Varkonyi-Gasic et al. 2013; Voogd et al. 2017; Herath et al. 2022), flesh color (Peng et al. 2019; Wang et al. 2019, 2022; Shu et al. 2023) and ascorbic acid (ASA) content (Liu et al. 2022, 2023b) have been elucidated. At present, the kiwifruit industry chain is facing unprecedented challenges, such as canker disease caused by phytopathogen *Pseudomonas syringae* pv. *actinidiae* (Psa), post-harvest storage diseases and abiotic stress. Therefore, further exploring distinct resistant germplasm resources is crucial to promote the development of kiwifruit genomics and functional genomics, and we are pleased to present a special collection named “Recent advances of kiwifruit genome and genetic transformation” to reflect the advances.

The Recent advances of kiwifruit genome and genetic transformation special collection contains five research articles that cover several aspects of the genome study, ranging from high-quality genome assembly, gene function identification and development of cutting-edge gene editing technology in kiwifruit.

Two cases of studies refer to improved quality of the genome assemblies using additional elite *A. eriantha* accessions. Compared to the *A. chinensis*, the *A. eriantha* has a relatively shorter juvenile period, exceptionally high ascorbic acid (ASA) content and strong Psa resistance, providing a valuable resource for both cross breeding and genetic improvement. Yao et al. (2022) used the third-generation sequencing technology to assemble the genome of a wild accession of *A. eriantha*, achieving a significantly improved assembly quality as compared to the previous version. This case study revealed structural variations between *A. chinensis* and *A. eriantha* associated with ASA synthesis and disease resistance pathways. Meanwhile, this work implicated introgression hybridization could contribute to the complex relationship between *A. eriantha* and other sequenced kiwifruit taxa and offer valuable genetic resource for breeding applications. Wang et al. (2023a) employed a comprehensive approach combining PacBio HiFi, ONT Ultra-long reads sequencing and Hi-C sequencing technologies to decipher the haplotype-phased genome of the *A. eriantha* cv. ‘MiDao 31’. This work has achieved gap-free, telomere-to-telomere and haplotype-resolved genomes of *A. eriantha* for the first time. In addition, the positions and sequence features of telomeres and centromeres were comprehensively predicted using a bioinformatics approach. This case study promotes the genome quality of *A. eriantha* up to the highest level, laying a solid foundation for further functional genomics research and genetic improvement of horticultural traits in kiwifruit.

The third case study deals with haplotype-resolved assembly of a complex genome. The *A. arguta*, also known as hardy kiwifruit or kiwi berry, is one of the most widely distributed kiwifruit species and exhibits excellent cold and Psa resistance. The ploidy level of *A. arguta* is highly complex, including diploid, tetraploid, hexaploid, octoploid, and decaploid, and majority of the cultivated varieties are tetraploids. Using third-generation sequencing technologies, Zhang et al. (2024) assembled the genome of an autotetraploid *A. arguta* cv. ‘Longcheng 2’ and successfully phased the homologous chromosomes into four sets of haplotypes. By analyzing synonymous substitution rates (K_s_) of both allelic and paralogous gene pairs across the assembled haplotypic individuals, the tetraploidization event in *A. arguta* is estimated to have occurred approximately 1.03 million years ago (MYA). In addition, the authors analyzed the nucleotide-binding domain and leucine-rich repeat (NBS-LRR) and C-repeat binding factor (CBF) gene families in *A. chinensis*, *A. eriantha* and *A. arguta*. They found that the expansion of these two gene families coming after tetraploidization may contribute to the enhanced ability of immune responses or environmental adaptability. Finally, through post-harvest transcriptomic analysis of the fruit, the authors used weighted gene co-expression network analysis (WGCNA) to identify transcription factors regulating the texture of *A. arguta*. This case study represents the first case of successfully decoding the complex genome of an autotetraploid *A. arguta*, shedding lights to understanding complex genome structures and facilitating functional genomics dissection and breeding not only for kiwifruit but also for other crops.

The fourth case study is concerned with functional identification of terpene synthase (TPS) gene family in kiwifruit. Volatile terpenes are one of the primary influencing factors on kiwifruit flavour, also playing a crucial role in attracting pollinating insects, repelling insects and defending against pathogen attacks. Wang et al. (2023b) conducted a comprehensive analysis of TPS gene family annotated from cv. ‘Red5’ genome (*A. chinensis*). As a result, 22 TPS gene models were annotated, of which 15 encode full-length TPS proteins and 13 TPS genes are accounted for the major terpene volatile compounds derived from the different tissues upon various stimuli. Treatment with methyl jasmonate (MeJA) enhanced the expression of defense-related TPS gene subgroups in leaves and fruit, stimulating terpene release. Six TPS genes were activated in response to leaf herbivory by the economically important insect pest *Ctenopseustis obliquana*, and emission of (*E*)- and (*Z*)-nerolidol was closely associated with herbivory. This case study provides insight into further understanding the overlapping biological and ecological roles of terpenes in *Actinidia* and other horticultural crops.

The fifth case study is the development of an advanced gene editing system and its utilization in gene function dissection in kiwifruit. The genetic transformation and gene editing of kiwifruit are particularly challenging and time-consuming. Li et al. (2024) have developed a rapid and efficient marker-free transformation and gene editing system for kiwifruit mediated by *Agrobacterium rhizogenes*. Additionally, they have devised a method to remove the root tips, which significantly promotes the regeneration efficiency of transgenic hairy roots. Through CRISPR/Cas9 gene editing mediated by *A. rhizogenes*, the editing efficiencies of *CEN4* and *AeCBL3* reached 55% and 50%, respectively. This method has been successfully applied to stably genetic transformation in both *A. chinensis* and *A. eriantha*. Using this system, the authors investigated the formation mechanism of calcium oxalate in kiwifruit. Overexpression of *AeCBL3* is shown to promote the formation of CaOx crystals, while knocking it out via CRISPR/Cas9 significantly impairs crystal formation in kiwifruit. In summary, this work has established a rapid, non-destructive transformation and highly efficient CRISPR/Cas9 gene editing system for various *Actinidia* genomes and will play a critical role in accelerating the exploration of functional genes associated with important horticultural traits.

In summary, the special collection on “Recent advances of kiwifruit genome and genetic transformation” represents fore frontiers in this field, and will be excellent references for researchers and a textbook for advanced trainees.
